# Prediction of Hanwoo Cattle Phenotypes from Genotypes Using Machine Learning Methods

**DOI:** 10.3390/ani11072066

**Published:** 2021-07-11

**Authors:** Swati Srivastava, Bryan Irvine Lopez, Himansu Kumar, Myoungjin Jang, Han-Ha Chai, Woncheoul Park, Jong-Eun Park, Dajeong Lim

**Affiliations:** Division of Animal Genomics and Bioinformatics, National Institute of Animal Science, Rural Development Administration, Wanju 55365, Korea; swati051@gmail.com (S.S.); irvinelopez@korea.kr (B.I.L.); himanshu.genetics@gmail.com (H.K.); minijmj@naver.com (M.J.); hanha@korea.kr (H.-H.C.); wcpark1982@korea.kr (W.P.)

**Keywords:** genomic prediction, machine learning, Hanwoo

## Abstract

**Simple Summary:**

Machine learning has been extensively used in analyzing big data and in conditions where the number of parameters is much bigger than the number of observations. Recently, there have been an increasing number of successful applications of machine learning in genomic prediction as this method makes weaker assumptions, is capable of dealing with the dimensionality problem, and can be more flexible for describing complex relationships. In this study, we evaluated the predictive ability of three machine learning methods, namely, random forest (RF), extreme gradient boosting (XGB), and support vector machine (SVM), when predicting the carcass traits of Hanwoo cattle. These machine learning algorithms were compared with the standard linear method (GBLUP). Our results revealed that XGB method had the best predictive correlation for carcass weight and marbling score. Meanwhile, the best predictive correlation for backfat thickness and eye muscle area was delivered by GBLUP. Moreover, in terms of mean squared error (MSE) of prediction, GBLUP delivered the lowest MSE value for all traits.

**Abstract:**

Hanwoo was originally raised for draft purposes, but the increase in local demand for red meat turned that purpose into full-scale meat-type cattle rearing; it is now considered one of the most economically important species and a vital food source for Koreans. The application of genomic selection in Hanwoo breeding programs in recent years was expected to lead to higher genetic progress. However, better statistical methods that can improve the genomic prediction accuracy are required. Hence, this study aimed to compare the predictive performance of three machine learning methods, namely, random forest (RF), extreme gradient boosting method (XGB), and support vector machine (SVM), when predicting the carcass weight (CWT), marbling score (MS), backfat thickness (BFT) and eye muscle area (EMA). Phenotypic and genotypic data (53,866 SNPs) from 7324 commercial Hanwoo cattle that were slaughtered at the age of around 30 months were used. The results showed that the boosting method XGB showed the highest predictive correlation for CWT and MS, followed by GBLUP, SVM, and RF. Meanwhile, the best predictive correlation for BFT and EMA was delivered by GBLUP, followed by SVM, RF, and XGB. Although XGB presented the highest predictive correlations for some traits, we did not find an advantage of XGB or any machine learning methods over GBLUP according to the mean squared error of prediction. Thus, we still recommend the use of GBLUP in the prediction of genomic breeding values for carcass traits in Hanwoo cattle.

## 1. Introduction

The Korean native cattle (Hanwoo) was originally raised for draft purposes, but the increase in local demand for red meat turned that purpose into full-scale meat-type cattle rearing; it is now considered one of the foremost economically important species and a vital food source for Koreans [1]. This breed has been subjected to intensive selection for particular meat quality and production attributes over the past few decades; thus, a dramatic improvement has been obtained in terms of carcass weight and rib eye area [2]. The application of genomic selection in Hanwoo breeding schemes in recent years was expected to lead to higher genetic progress. In beef cattle, genomic prediction offers great promise to predict total genetic value of selection candidates, especially for traits that cannot be measured directly, such as carcass traits. The successful application of genomic selection relies on the accuracy of genomic estimated breeding values (GEBVs), which are mostly determined using estimation methods.

Up to date, different genomic prediction methods based on linear models have been developed, such as genomic best linear unbiased prediction (GBLUP) [3], single-step GBLUP [4], the Bayesian alphabet (Bayes A, Bayes B, Bayes Cπ, and BayesR) [5,6,7], and the ridge regression BLUP (RR-BLUP) [8]. However, these statistical methods typically make strong assumptions about functional forms and the statistical distribution of marker effects. Thus, these methods pose statistical challenges related to high-dimensional genomic data and have difficulty capturing complex relationships between genotypes and phenotypes such as genotype-by-environment-by-trait interactions [9,10]. Recently, there have been an increasing number of successful applications of machine learning in genomic prediction [11]. These machine learning approaches make weaker assumptions, are capable of dealing with the dimensionality problem, and can be more flexible for describing complex relationships [12].

Machine learning methods, such as random forest (RF) [13], boosting [14], and support vector machine (SVM) [15], provide an appealing alternative to conventional statistical methods for genomic prediction of quantitative traits. They may provide an importance measure of predictor variables (SNPs) on a given trait and good predictive performance. RF and boosting are independent of model specification and, hence, may account for non-additive effects. Moreover, SVM is powerful at recognizing subtle patterns in complex datasets [15]. Recently, the extreme gradient boosting (XGB) [16] method was introduced with a similar principle to the gradient boosting method but with increased speed and less overfitting. Several studies using RF, XGB, and SVM have been used for genomic-based prediction in animal and plants [11,17,18].

The objective of this study was to compare the predictive performance of three machine learning methods, namely, RF, XGB, and SVM, with the conventional genomic prediction model (GBLUP) when predicting the carcass weight (CWT), marbling score (MS), backfat thickness (BFT) and eye muscle area (EMA) of Hanwoo cattle. Comparisons in terms of predictive correlation and mean squared error were used as metrics.

## 2. Materials and Methods

### 2.1. Data

The data on four carcass traits were collected from 7234 Hanwoo cattle slaughtered at the age of around 30 months. Carcass weight (CWT), marbling score (MS), backfat thickness (BFT), and eye muscle area (EMA) were the traits under study. The animals in this study were produced through the purebred mating system done using artificial insemination of semen collected from bulls initially selected on the basis of their performance and progeny carcass traits. All of these animals were gathered from different herds in nine provinces across South Korea. The ethics approval for this study was given by the Animal Care and Use Committee of the National Institute of Animal Science, Rural Development Administration, Korea (2018-293). The descriptive statistics for each trait are presented in Table 1. Phenotypic records were adjusted for fixed effects as a function of a univariate analysis using the PREDICTF90 software package [19] in a pedigree-based model described in our previous study [20]. Briefly, the fixed effects that were used for all traits were herds, year-month of birth, year-month of slaughter, and slaughter place, along with sex and age as covariates.

A total of 7324 animals were genotyped for 53,866 SNPs using the customized Hanwoo 50K SNP Chip (Illumina, Korea) according to the manufacturer’s protocol. The genomic DNA was quantified from tissue samples using the DNeasy Blood and Tissue Kit (Qiagen, Valencia, CA, USA). The following threshold levels were applied for quality control using PLINK [21]: SNPs with minor allele frequency lower than 0.01, call rate lower than 0.90, Hardy–Weinberg disequilibrium with a *p*-value lower than 0.0000001, and situated on the sex chromosomes were removed from the genotype data. After quality control, 45,624 SNPs were retained for genomic prediction.

### 2.2. Statistical Methods

In this study, three machine learning algorithms, namely, random forest (RF), extreme gradient boosting (XGB), and support vector machine (SVM), were evaluated. These machine learning algorithms were compared with the standard linear method, GBLUP. The predictive performance of the different methods was assessed using a fivefold cross-validation scheme composed of five subpopulations that were randomly split into more or less equally sized groups. In cross-validation, each subpopulation (~1446) was given a chance to be used as the validation set and the other four subpopulations were used as the training set. The predictive correlation was calculated as a Pearson correlation between predicted and observed phenotypes. Furthermore, the mean squared errors of prediction were calculated. 

#### 2.2.1. Genomic Best Linear Unbiased Prediction (GBLUP)

The general animal model could be expressed as

$$y=1\mu +s_{i}\alpha_{i}+Zg+e,$$

where **y** is a vector of observed phenotypes, **μ** is the overall mean, **1** is a vector of ones, **s_i_** is a vector of genotypes for SNP_i_ (coded as 0, 1, or 2), $\alpha_{i}$ is the size of the effect of the marker (allele substitution effect), **g** is a vector of the genomic breeding values of all individuals $\left[g\;\sim \;N\left(0,\;G\sigma_{g}^{2}\right)\;\right],$ where $\sigma_{g}^{2}$ is the additive genetic variance and **G** is the marker-based genomic relationship matrix [3], **Z** is an incidence matrix linking **g** to **y,** and **e** is the vector of random residual effect $\left[e\;\sim \;N\left(0,\;I\sigma_{e}^{2}\right)\;\right].$ The software MTG2 version 2.21 [22] was used to estimate variance components with restricted maximum likelihood (REML) and to calculate the genomic breeding values (GEBVs).

#### 2.2.2. Random Forest (RF)

Random forest is a type of bagging method which is also known as bootstrap aggregating, and it was first proposed by Breiman [13]. It is a compilation of uncorrelated forests of trees whose prediction is more accurate than that of any single or group of trees. It estimates and fits a number of decision trees on various subsamples of the dataset and then uses their average to improve predictive accuracy and control overfitting. This method involves feature selection, generating predictors with the least correlation [13]. Therefore, the initial step was to identify significant features in our data. For this, the feature selection library present in scikit-learn was used. In order to find the best estimator, random search by cross-validation was used on hyperparameters. These parameters were ‘n_estimator’ used to find number of trees in the forest, ‘max_features’ used to find the maximum number of features considered for splitting a node, ‘max_depth’ used to find the maximum number of levels in each tree, ‘min_samples_split’ used to find the minimum number of data points placed in a node before the node was split, and ‘bootstrap’ used for sampling data points (with or without replacement). This “fit” and “score” methods with parameters used in this approach were optimized by cross-validation search. The model parameters used in this study for each trait are shown in Table 2. These parameters were selected by the randomsearch function in scikit-learn using Python [23] and were used to build the RandomForestRegressor model.

#### 2.2.3. Extreme Gradient Boosting Method (XGB)

The extreme gradient boosting (XGB) method [16] is a kind of ensemble machine learning algorithm that converts weak learners into strong learners, either for regression or for classification problems to reduce bias in supervised learning. This method applies the principle of boosting weak learners (CARTs generally) using the gradient descent architecture. It controls overfitting and can reduce prediction errors by utilizing more regularized model formation. The feature selection method was also used in this method to identify significant features through scikit-learn. Scores generated by this method generally gain value, generated by the decrease in prediction error of the objective function to a split node in a tree. Some of the important parameters considered to build the model were ‘booster’ to determine the type of learner, either its tree or linear function, ‘eta’ analogous to learning rate, ‘min_child_weight’ to determine the minimum sum of weights, ‘max_depth’ to find the maximum number of levels in each tree, ‘max_leaf_nodes’ as the maximum number of terminal nodes, and ‘gamma’ reflecting the minimum loss function. The scikit-learn XGBRegressor [23] in Python was used for model construction. In this study, booster was selected as ‘gbtree’ (i.e., tree based model), ‘eta’ was kept as 0.3, ‘min_child_weight’ was kept as 1 (minimum sum of weights), maximum depth was selected as 6, maximum leaf nodes were selected as 6 (maximum number of terminal nodes), and gamma was kept as 0 (i.e., minimum loss function).

#### 2.2.4. Support Vector Machine (SVM)

The support vector machine (SVM) is a supervised regression method that supports linear and nonlinear regression. Generally, SVM is used for classification or regression problems. It works on the basis of enlarging the feature space using various kernels such as linear, polynomial, and sigmoid Gaussian RBF (radial basis function).Linear kernels are mainly used for linear problems, whereas RBF kernels are used for nonlinear problems. A thorough guide and review on this method can be found in Smola and Schölkopf [24]. In this study, we applied the RBF kernel for building the model using epsilon-support vector regression. ‘StandardScaler’ and RandomForestRegressor models were built using scikit-learn [23], considering all features.

## 3. Results and Discussion

### 3.1. Genetic Parameters

Variance components and heritability estimates for each trait are presented in Table 3. Overall, the estimate of heritability for carcass traits in Hanwoo cattle was medium to high. Heritability estimates for CWT, MS, BFT, and EMA were 0.38, 0.44, 0.36, and 0.35, respectively. The standard errors of estimated heritability for all traits were 0.02. The estimated heritability for each trait in this work was lower, higher, or in the range of previously reported estimates [25,26,27]. The observed differences between the estimates in this study and previous works may have been due to the population structure, number of records, fixed effects, and information (pedigree and/or genomic) used.

### 3.2. Genomic Prediction

**Figure 1:**
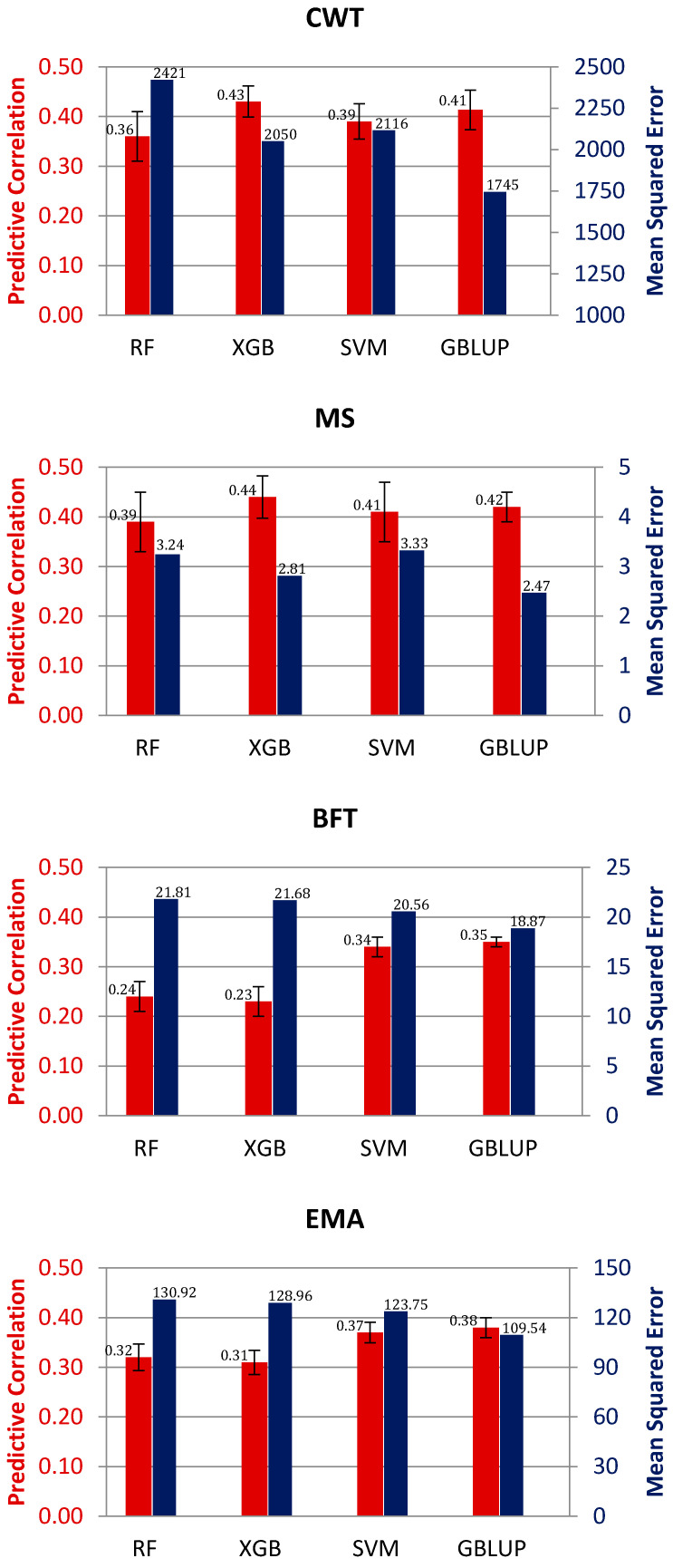
Predictive correlation (red color) and mean squared error (blue color) of prediction obtained using different statistical methods for carcass weight (CWT), marbling score (MS), backfat thickness (BFT), and eye muscle area (EMA). RF: random forest, XGB: extreme gradient boosting, SVM: support vector machine, GBLUP: genomic best linear unbiased prediction.

Figure 1 displays the predictive correlation and mean squared error (MSE) using GBLUP and three machine learning methods, namely, RF, XGB, and SVM, for four carcass traits of Hanwoo cattle. The results showed that the boosting method XGB showed the highest predictive correlation for CWT and MS, followed by GBLUP, SVM, and RF. Meanwhile, the largest predictive correlation for BFT and EMA was delivered by GBLUP, followed by SVM, RF, and XGB. The average correlations using GBLUP for CWT, MS, BFT, and EMA were 0.41, 0.42, 0.35, and 0.38, while they were 0.43, 0.44, 0.23, and 0.31 when using XGB, respectively. Correspondingly, the mean correlations using SVM were 0.39, 0.34, 0.42, and 0.37, and those using RF were 0.36, 0.39, 0.24, and 0.32, respectively. Predictive correlation is a common and simple way of measuring predictive performance, but MSE is a preferred parameter because it takes into account both prediction bias and variance. In this sense, GBLUP delivered the lowest MSE for all traits among methods. Meanwhile, among the machine learning methods, the lowest MSE for CWT and MS was achieved with XGB, whereas the best performer was SVM for BFT and EMA.

The random forest method has been used in many genomic prediction studies. González-Recio and Forni [11] compared the RF method with Bayes A and Bayesian LASSO using simulated discrete data and disease resistance data in pigs. They reported that RF outperformed those methods, with better classification performance within and across datasets. In this study, the RF method delivered the lowest predictive ability among the methods in general. This is consistent with the results of Abdollahi-Arpanahi et al. [28], who reported that GBLUP and Bayes B had a higher predictive correlation and lower MSE value than RF using a real dataset of Holstein bulls with sire conception rate records, genotyped for 58k SNPs. Moreover, Ogutu et al. [29] reported that RR-BLUP, boosting, and SVM methods had higher predictive correlations than RF in a study using simulation.

The SVM method is a popular machine learning algorithm used in genome-enabled prediction due to its capability to handle potential nonlinearity between features and target traits in both animals and plants [17,30,31]. Previous studies have shown contrasting results regarding the predictive performance of SVM over linear models [17,29,32,33]. In this study, the predictive correlation of the SVM model ranked second in two traits, and the difference in performance with the GBLUP model was small for all traits (Figure 1). Zhao et al. [17] compared the predictive ability of SVM, GBLUP, and BayesR methods using pig datasets. They reported that the prediction accuracy was very similar among methods. Meanwhile, Tusell et al. [33] showed that the SVM models could outperform the conventional GBLUP in predicting average residual feed intake and average daily gain crossbred performances from purebred sire genotypes. 

Among machine learning methods, only the boosting method XGB outperformed GBLUP for some traits (CWT and MS) in terms of predictive correlation, as shown in Figure 1. Previous studies showed that the boosting method had a better predictive performance than other machine learning methods such as RF, SVM, and convolutional neural networks [28,29]. This could be due to its efficient ‘weak learner’ algorithm and stepwise assembling method with sequential learning to build the model, unlike parallel learning in the case of RF (bagging method). Another potential reason for such a better predictive performance could be that the boosting method trees are constructed following a greedy search algorithm or optimizing an objective function (e.g., ranking and Poisson regression), whereas RF constructs trees independently, using random samples of data. 

Among machine learning methods, there is no universal prediction model. Predictive ability depends on the trait and is affected by many factors. In the machine learning field, the “no free lunch theorem” [34] states that there is no algorithm uniformly better for all species and traits that will work optimally for each problem each time. Thus, the best method may be case-dependent, and an initial evaluation of different methods is recommended to deal with a particular problem.

## 4. Conclusions

Our results indicated that machine learning method XGB had the best predictive correlation for CWT and MS. Meanwhile, the highest predictive correlation for BFT and EMA was achieved by GBLUP. Although XGB presented higher predictive correlations for some traits, we did not find an advantage of XGB or any other machine learning method over GBLUP in terms of mean squared error of prediction. Therefore, we still recommend the use of the conventional statistical method GBLUP in the prediction of genomic breeding values for carcass traits in Hanwoo cattle.

## Figures and Tables

**Table 1:** Descriptive statistics for carcass traits of Hanwoo cattle.

Trait	Mean	SD	Min	Max
CWT (in kg)	439.33	49.47	159	645
MS (1–9)	5.99	1.84	1	9
BFT (in mm)	14.24	4.78	1	45
EMA (in cm^2^)	96.15	11.96	35	155

SD, standard deviation; CWT, carcass weight; MS, marbling score; BFT, backfat thickness; EMA, eye muscle area.

**Table 2:** Parameters used to build model for RF method for each trait.

Trait	N_Estimator	Criterion	Max_Features	Min_Samples_Leaf	Min_Samples_Split	Max_Depth	Bootstrap
CWT	400	MSE	auto	4	10	70	TRUE
MS	600	MSE	auto	4	2	40	TRUE
BFT	2000	MSE	auto	2	2	90	TRUE
EMA	1400	MSE	auto	4	2	100	TRUE

MSE, mean square error; CWT, carcass weight; MS, marbling score; BFT, backfat thickness; EMA, eye muscle area.

**Table 3:** Additive genetic variance (**σ^2^_a_**), residual variance (**σ^2^_e_**), phenotypic variance (**σ^2^_p_**), and heritability estimates (**h^2^**) for carcass traits of Hanwoo cattle.

Trait	σ^2^_a_	σ^2^_e_	σ^2^_p_	h^2^
CWT	773.00	1266.48	2039.48	0.38
MS	1.29	1.67	2.96	0.44
BFT	7.77	14.04	21.81	0.36
EMA	43.80	81.89	125.69	0.35

CWT, carcass weight; MS, marbling score; BFT, backfat thickness; EMA, eye muscle area.
